# Supplementary material for: Optimized Traffic Light System with AIC and Application to the 2021 M6.7 Yangbi Earthquake Sequence
Source: Entropy (Basel). 2023 May 6;25(5):759. doi: 10.3390/e25050759 (PMC10217739; doi:10.3390/e25050759)
Supplement: Supplementary file 1 [file entropy-25-00759-s001.zip › entropy-2340866-supplementary.pdf]

Supplemental material for

Optimized traffic light system with AIC and application to the 2021 M6.7 Yangbi earthquake sequence

Rui Wang[1], Ying Chang[2,*] Peng Han[1, 3], Miao Miao[1, 3], Zhiyi Zeng[1], Haixia Shi[4], Danning Li[5], Lifang Liu[5], and Youjin Su[5]

1 Department of Earth and Space Science, Southern University of Science and Technology, Shenzhen 518055, China; 11930870@mail.sustech.edu.cn (R.W.); hanp@sustech.edu.cn (P.H.); miaom@sustech.edu.cn (M.M.); 11930855@mail.sustech.edu.cn (Z.Z.)

2 Institute of Mining Engineering, BGRIMM Technology group, Beijing 100160, China; changy@bgrimm.com (Y.C.)

3 Guangdong Provincial Key Laboratory of Geophysical High-resolution Imaging Technology, Southern Uni-versity of Science and Technology, Shenzhen 518055, China.

4 China Earthquake Networks Center, Beijing 100045, China; shihaixia08@seis.ac.cn (H.S.)

5 Earthquake Administration of Yunnan Province, Kunming 650224, China; zuni_2001@163.com (D.L.); li-fang_l@sina.com (L.L.); suyoujin0818@sina.com (Y.S.)

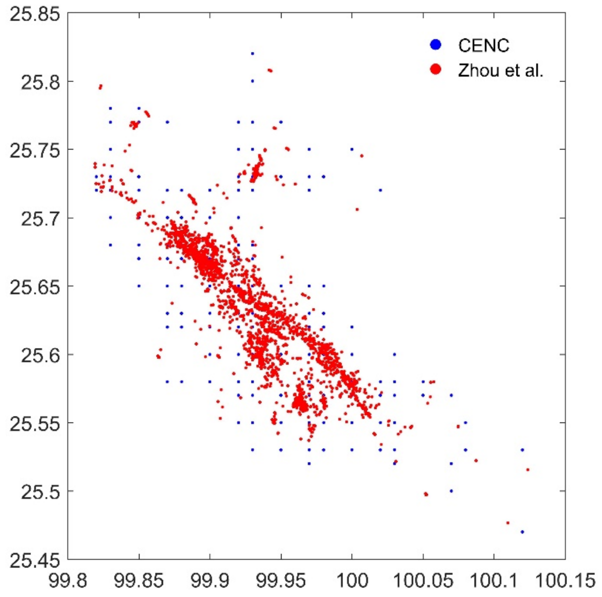

(a)

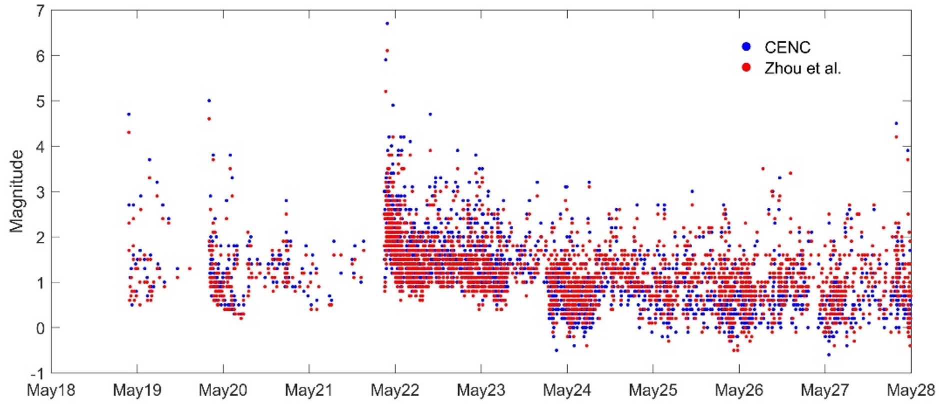

(b)

Figure S1 The distribution of the same events of catalogs of CENC and Zhou et al. the blue and red points are earthquakes in catalog of CENC and Zhou et al. respectively. (a) Spatial distribution of the same events. (b) Temporal distribution of the same events.

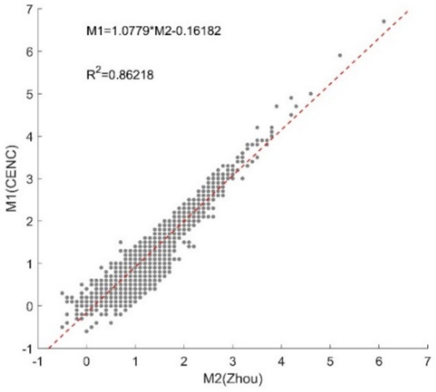

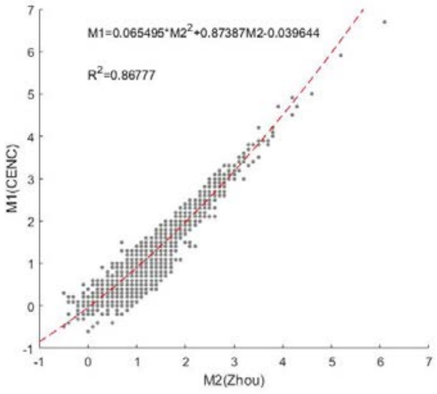

(a) (b)

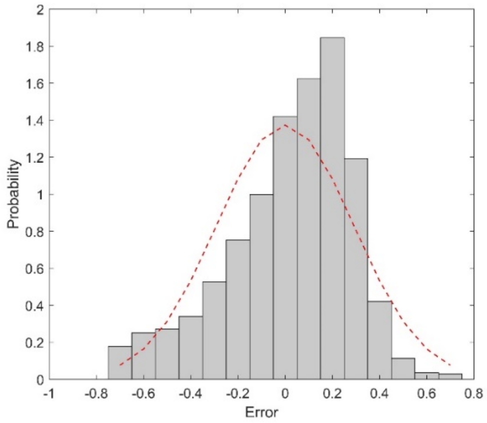

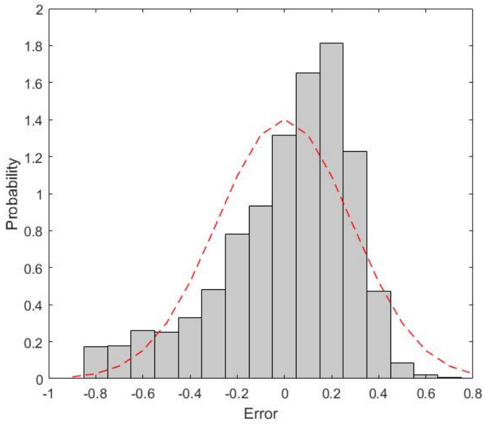

(c) (d)

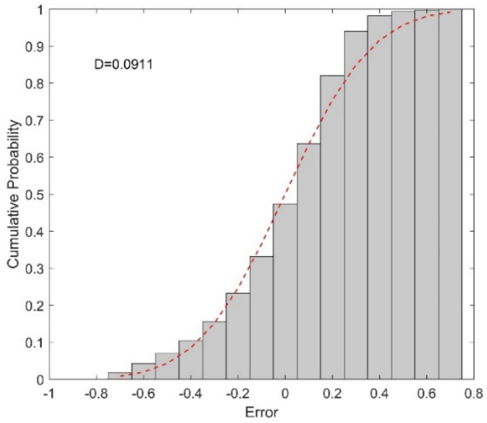

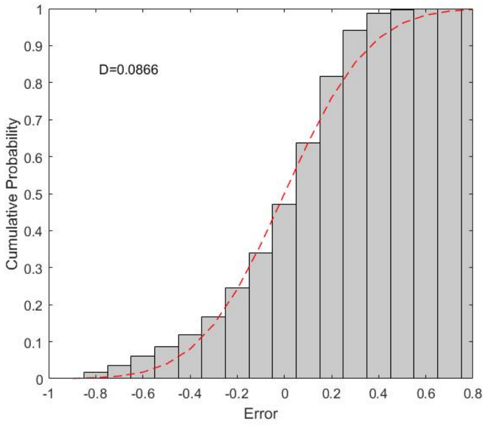

(e) (f)

Figure S2 The linear regression and error distribution. (a) The magnitude of Zhou et al.-CENC, 1 order linear regression results (red dotted line), degree of fitting (R^2) and

coefficients. (b) The magnitude of Zhou et al.-CENC, 2 order linear regression results (red dotted line), degree of fitting (R^2) and coefficients. (c)The error distribution of magnitude in (a) and normal distribution. (d) The error distribution of magnitude in (b) and normal distribution. (e)The cumulative error distribution of magnitude in (a) and cumulative normal distribution. (f)The cumulative error distribution of magnitude in (b) and cumulative normal distribution.

We performed a 1st and 2nd order (shown in supplementary materials) linear regression between the same events of CENC and Zhou et al. catalogs (Zhou, Zhou, and Zhuang 2018) (shown in FigureS1), as shown in FigureS2. The fitting degrees of 1st and 2nd order are, respectively, 0.86218 and 0.86777, between which is small difference. We choose the 1st order linear regression coefficients to convert the magnitude of catalog of Zhou et al. and add it to the CENC catalog. About the events in both catalogs, we keep the magnitude of primary catalog and other data of Zhou et al.'s catalog.

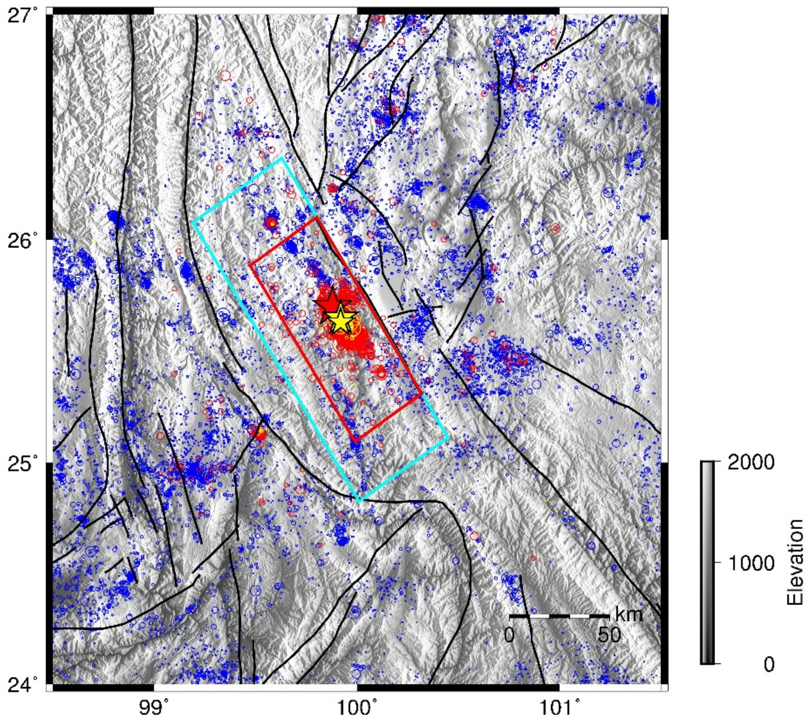

(a)

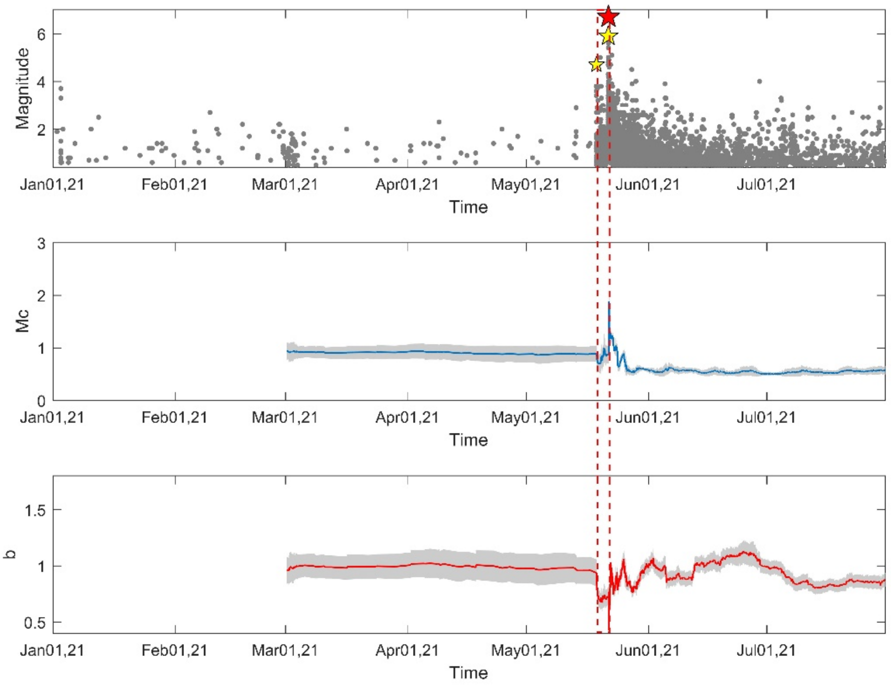

(b)

Figure S3 (a) The new study area of temporal variation of b value (the red edge rectangle). (b) The temporal variation of b value in new study area.
